# A Case of Facial Lipoatrophy Secondary to Lupus Profundus Managed with Lipofilling Technique

**DOI:** 10.1155/2012/720518

**Published:** 2012-12-04

**Authors:** Luigi Valdatta, Mario Cherubino, Federico Tamborini, Igor Pellegatta, Francesca Maggiulli

**Affiliations:** U.O. di Chirurgia Plastica e Ricostruttiva, Ospedale di Circolo e Fondazione Macchi, Università degli Studi dell'Insubria, Viale Borri 57, 21100 Varese, Italy

## Abstract

Facial lipoatrophy is one of the most difficult complication in the patients with lupus profundus. In this paper, we present a case of a 55-year-old woman affected by lupus profundus, with a grade V lipoatrophy, treated with lipofilling technique. No complications were observed and results at 12 months were stable, natural, and symmetric.

## 1. Introduction

**Figure 1:**
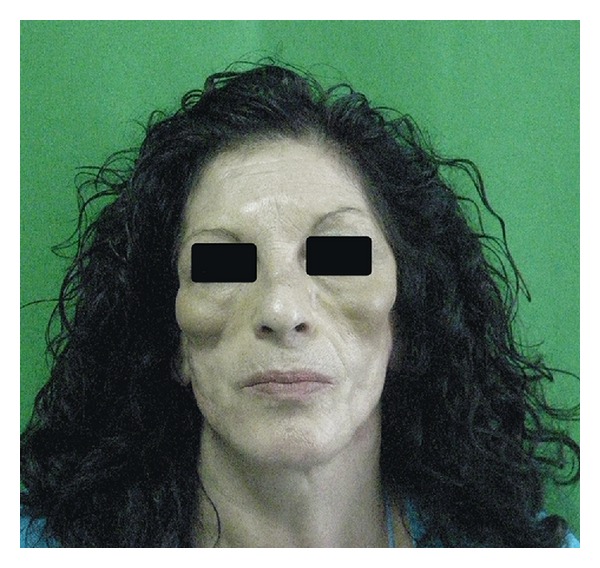
Initial condition of the patient.

Lupus profundus [1–6] is a clinical condition characterized by subcutaneous nodular lesions localized at the face, arms, legs, trunk, and abdominal region (see Figure 1). Lipoatrophy follows the nodular stage after its resolution. Lupus profundus incidence is between 1% and 3% in patient with LES. Autoimmunitary etiology is confirmed by low complement levels and presence of antinucleus antibody. Manifold complications are possible in lupus profundus: lipoatrophy, alopecia [7], enophthalmos [8, 9], central retinal artery occlusion [10], mastitis [11], thrombophlebitis [12], and proptosis [13]. Despite its benignity, lipoatrophy is psychologically important in everyday life. Facial lipoatrophy can be considered following a grading scale [14] from first to fifth grade. Different strategies were proposed in order to solve this situation: nonabsorbable fillers such as polymethylacrylate [15] do not give striking results, and complications [16], as granulomatous “rubberizing” reaction, are rare but possible. Free flaps, especially anterolateral thigh flap [17] and temporal flap [18], are rarely used giving the difficulty of the surgical procedure and the unnatural results. Yoshimura's CAL technique [19] is a valid and quite stable option but requires adipose-derived stem cells extraction in laboratory, 7–10 days for cell culture, and a double step surgical procedure (fat extraction and fat injection). Authors report their experience regarding a case of patient with Lupus Profundus treated with lipofilling technique [20–22].

## 2. Matherials and Methods

A 55-year-old patient suffering from lupus panniculitis, treated with hydroxychloroquine, methotrexate, and metilprednisone, showed clinical signs of lipoatrophy in facial, abdominal, and mammary areas. Patient was submitted to three surgical procedures of lipofilling with Coleman's technique [23] at 6-month distance among every step. First adipose graft was 12 cc, second 15 cc, and third 18 cc, picked from abdominal fat. Submalar, parotideal, perioral, and mandibular sites was grafted with adipose tissue, using 9 French microcannules. Only one hole for each side of the face was necessary to introduce the graft. Every procedure was performed in outpatient surgery and in local anesthesia with sedation. 

## 3. Results

**Figure 2:**
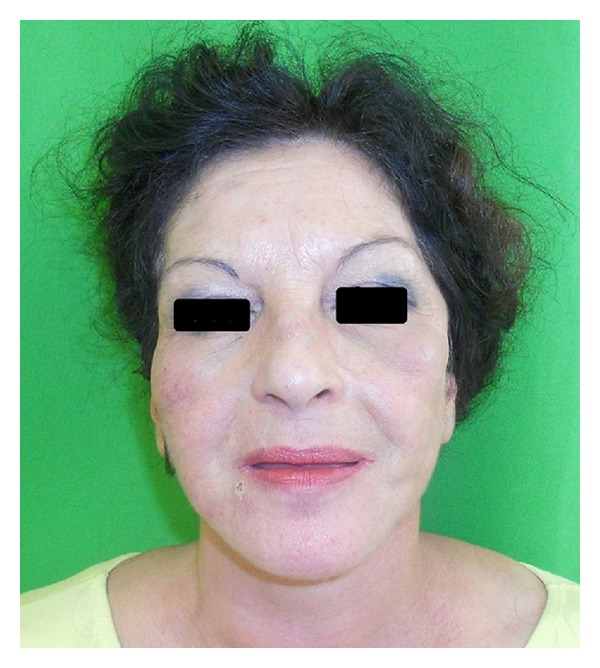
Final result after three lipofilling procedures at 12-month followup.

No complications due to infections or surgical technique were observed after each procedure. A mild intolerance to the antibiotic therapy was easily solved. 12-month evaluation after third reconstructive step showed the stability of the result and the emptiness localized in the submalar and parotideal region was totally filled (see Figure 2). Also the aspect of typical cutaneous lesions of lupus syndrome was improved. The first of three grafts did not supply an acceptable result as because of copious smoking by the patient during immediate postoperatively period. Final result was natural and symmetric.

## 4. Discussion

Facial lipoatrophy is an important issue for the aesthetic acceptance of a patient affected by lupus profundus. In our case a triple lipofilling procedure has given stable results at 12-month followup. Longer followup would be necessary to prove the persistence of the results.

Despite its rarity, severe complications of lipofilling in the treatment of facial lipoatrophy are reported [24]. The impairment of facial nerve may represent one of the problems due to surgical technique.

Fat grafting should be examined as option in management of three-dimensional defects of facial lipoatrophy: the minimal invasivity, the simplicity of the surgical procedure, the fast convalescence, and the possibility of modulating the quantity of the graft are the main benefits of this option.

In our experience one of the most essential factors in the persistence of the graft was patient's smoking habit. We believe that absolute abstinence in smoking in the months after the surgery would have dramatically improved the final results and decreased the number of procedures needed. Moreover, we repute that, as demonstrated in breast augmentation [25], new automated devices extracting adipose stem cells would improve the graft persistence.

## 5. Conclusions

Lupic panniculitis is a relatively rare condition that causes facial lipoatrophy. Different strategies were adopted in order to fill the soft tissue defect. Nonabsorbable filler and free flaps are two of the options available. The management of the above-mentioned case provided good results. No complications were observed and the period of healing was minimal. Photographic evaluation demonstrates the persistence of adipose tissue and patient satisfaction degree was excellent. Hence, we believe that lipofilling would represent a simple, cheap, and fast method to face an important complication of lupus profundus, as facial lipoatrophy.
